# Smartphone-Guided Algorithms for Use by Community Volunteers to Screen and Refer People With Eye Problems in Trans Nzoia County, Kenya: Development and Validation Study

**DOI:** 10.2196/16345

**Published:** 2020-06-19

**Authors:** Hillary Rono, Andrew Bastawrous, David Macleod, Cosmas Bunywera, Ronald Mamboleo, Emmanuel Wanjala, Matthew Burton

**Affiliations:** 1 Clinical Research Department International Centre for Eye Health London School of Hygiene & Tropical Medicine London United Kingdom; 2 Kitale County and Referral Hospital Kitale Kenya; 3 Peek Vision Foundation London United Kingdom; 4 Medical Research Council Tropical Epidemiology Group London School of Hygiene & Tropical Medicine London United Kingdom; 5 Moorfields Eye Hospital NHS Trust London United Kingdom

**Keywords:** visual impairment, algorithms, mobile phone, screening, mHealth, sensitivity, specificity

## Abstract

**Background:**

The provision of eye care services is currently insufficient to meet the requirements of eye care. Many people remain unnecessarily visually impaired or at risk of becoming so because of treatable or preventable eye conditions. A lack of access and awareness of services is, in large part, a key barrier to handle this unmet need.

**Objective:**

This study aimed to assess whether utilizing novel smartphone-based clinical algorithms can task-shift eye screening to community volunteers (CVs) to accurately identify and refer patients to primary eye care services. In particular, we developed the Peek Community Screening app and assessed its validity in making referral decisions for patients with eye problems.

**Methods:**

We developed a smartphone-based clinical algorithm (the Peek Community Screening app) using age, distance vision, near vision, and pain as referral criteria. We then compared CVs’ referral decisions using this app with those made by an experienced ophthalmic clinical officer (OCO), which was the reference standard. The same participants were assessed by a trained CV using the app and by an OCO using standard outreach equipment. The outcome was the proportion of all decisions that were correct when compared with that of the OCO.

**Results:**

The required sensitivity and specificity for the Peek Community Screening app were achieved after seven iterations. In the seventh iteration, the OCO identified referable eye problems in 65.9% (378/574) of the participants. CVs correctly identified 344 of 378 (sensitivity 91.0%; 95% CI 87.7%-93.7%) of the cases and correctly identified 153 of 196 (specificity 78.1%; 95% CI 71.6%-83.6%) cases as not having a referable eye problem. The positive predictive value was 88.9% (95% CI 85.3%-91.8%), and the negative predictive value was 81.8% (95% CI 75.5%-87.1%).

**Conclusions:**

Development of such an algorithm is feasible; however, it requires considerable effort and resources. CVs can accurately use the Peek Community Screening app to identify and refer people with eye problems. An iterative design process is necessary to ensure validity in the local context.

## Introduction

### Background

It is estimated that 216.6 million people globally are visually impaired (visual acuity in the better eye <6/18), and 36 million are blind (visual acuity in the better eye <3/60) [1]; about 90% of them live in low- and middle-income countries [2]. In sub-Saharan Africa, about 26 million people are visually impaired, and almost 6 million are blind [3].

The high prevalence of visual impairment (VI) is attributed to poverty and lack of access to eye services [4], shortages of health workers trained in eye care [5], and lack of awareness of the eye conditions they have [6]. Few countries in sub-Saharan Africa have reached the World Health Organization (WHO)–suggested ophthalmic cadre minimum targets of one ophthalmologist for 250,000 people to meet the surgical needs of population [7,8]. Some countries, especially in Africa, have trained midlevel personnel, including ophthalmic nurses and ophthalmic clinical officers (OCOs), to share key tasks and to compensate for the lack of ophthalmologists [9,10]. In those countries, they provide the bulk of eye care (including preventive, diagnostic, and referral services) in most rural and remote areas [11]. Generally, the few available eye health workers are concentrated in urban areas, further increasing the inequality in access to eye health care [7,12]. For example, in Trans Nzoia, a rural county in Kenya, with a population of 818,757 [13], the doctor to population ratio is 5.4 per 100,000, and the nurse to population ratio is 47 per 100,000 people [14]. This is lower than the recommended WHO minimum ratio of 230 per 100,000 population for any cadre [15].

An important strategy to improve access to eye care is task shifting, with redistribution of tasks within the health workforce, through clear referral criteria and management plans [16]. For example, guided task shifting through clinical algorithms defined as a text (flow chart) representing clinical decisions for guiding patient care [17] are a core part of the Integrated Management of Childhood Illness (IMCI) [18]. IMCI algorithms are effective in identifying pneumonia, gastroenteritis, measles, malaria, and malnutrition; however, eye conditions were not included [19]. Clinical algorithms have also been developed for use in eye care, although the accuracy of these algorithms has been variable. These include the *Edinburgh Red Eye Diagnostic Algorithm* to determine the correct ophthalmic diagnosis in a hospital by non–eye care nurses [20], and the *Edinburgh Visual Loss Algorithm* to assess the cause of visual loss by clinicians with no experience in ophthalmology [21]. Recently, the WHO developed and published clinical algorithms for primary health care (PHC) workers in Africa to assess patients with eye conditions; if proved acceptable, these algorithms could improve decision making at the PHC level [22].

Mobile health (mHealth) defined as the use of mobile and wireless technologies to support the achievement of health objectives is increasing and gaining acceptance [23,24]. There are a growing number of mHealth interventions for eye care. These include Peek Acuity, a smartphone or tablet app for measuring visual acuity [25]. A trial in primary schools in Kenya demonstrated that teachers could use Peek Acuity to detect VI (visual acuity <6/12) in school children who were aged 6 years or older [26]. This provided evidence that mHealth solutions could enable task shifting and improve access to eye health services.

In this study, we describe the process of developing and testing the Peek Community Screening app. A smartphone-based referral decision support algorithm designed to guide users to identify eye problems, which need referral using common eye signs and symptoms. To our knowledge, this is the first smartphone-based algorithm to aid referral of patients with eye problems from the community to primary eye care.

The target system users were community volunteers (CVs)—individuals who live in the community—and are selected by the community to represent them on issues of health [27]. Their roles include health promotion, referring cases to the nearest health facility, visiting homes to determine health status, and communication with household members [28,29]. They receive a short defined informal training that is relevant to their work.

**Figure 1:**
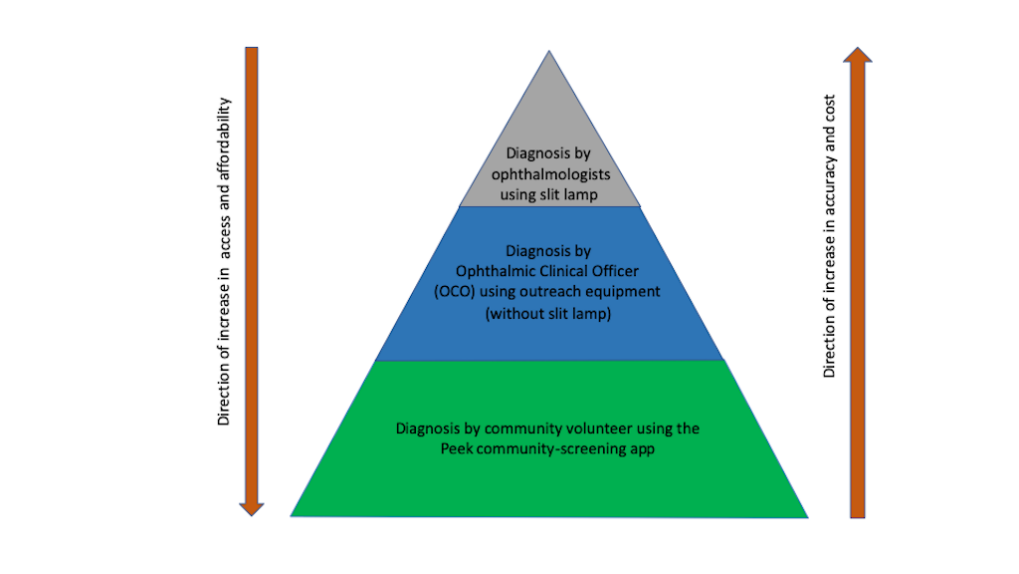
Conceptual framework for the various methods used to identify eye problems.

Most studies have used ophthalmologists as the reference standard [20-22]. OCOs have also been used in other studies [26,30]. In some countries where there are few ophthalmologists, OCOs provide most eye care services especially in rural areas [11]. On this basis, assessments by OCOs are acceptable. We chose OCOs because the majority of them work in rural areas (context where the app is used), they are the first contact for people with eye problems, and they have the relevant experience to make diagnoses and treatment decisions using available equipment in outreach settings. We developed a theoretical framework for assessing eye problems using principles from a framework used to train CVs to identify stroke in Pakistan (Figure 1) [31].

### Objectives

The aim of this study was to develop the Peek Community Screening app and assess its validity in making referral decisions for patients with eye problems. This paper outlines the development process and the results of using the app over a number of iterations, where the algorithm was altered to improve its performance, before settling on a final algorithm to be taken forward. We describe in detail the results for the final algorithm.

## Methods

### Ethics Approval

The approval was granted by the London School of Hygiene and Tropical Medicine Ethics Committee, the United Kingdom, and the Institutional Research and Ethics Committee in Moi University, Eldoret, Kenya. The study adhered to provisions of the Helsinki Declaration. Written informed consent was obtained from all participants.

### Development and Prevalidation Testing

We initially adopted the signs and symptoms used in a study that predicted eye conditions requiring referral in Rwanda, Madagascar, and Malawi [30], and incorporated the process used in developing the WHO clinical algorithms for PHC as a starting point for the design of our algorithms [22]. We adapted them to the environment and context for Trans Nzoia County for which the algorithms were to be used. The factors considered in making referral decisions were age, the presence of signs and symptoms of common eye problems, and visual acuity. Initially, decision trees were drawn on paper and tested informally on a small number of individuals in a hospital setting. In early tests, we observed low specificity, and incrementally changed the algorithm based on the observed results and clinical knowledge of the study authors.

From this formative work, we then developed guided questions and assessments for the CVs in order for them to be able to make referral decisions. Using the potential responses to the questions, we developed a workflow and decision matrix that were, then, translated into a digital-guided form operated on Android (Google LLC, Mountain View, CA) smartphones or tablets. The decision matrices (algorithms) were coded into a prototype app, the Peek Community Screening app, in collaboration with Peek Vision (London, UK) for use by the CVs.

We adopted a two-phase (hospital and community) prevalidation process to ensure that the final algorithm was accurate, relevant, and acceptable in this setting, and also to prepare the team adequately before the formal validation study [32]. On the basis of the clinical experience of the authors, we set the sensitivity of the algorithm to be no less than 90% and specificity above 75%. We selected and trained the CVs before commencing the prevalidation in the community setting.

Four CVs were purposefully selected from a pool of practicing CVs. A 3-day training of CVs, on how to use the Peek Community Screening app to identify and refer participants with eye problems, was conducted by two authors. Written guides, roleplays, and supervised practice sessions using consenting patients from the eye department were used for teaching purposes. Two CVs discontinued the training because of personal reasons while the remaining two CVs conducted all the validations.

To assess the consistency of CVs using the app, the same patients were independently examined by the lead author and by the two remaining CVs, all using the Peek Community Screening app to make an automated referral decision. We compared the referral decisions of the CVs with those of the lead author using the same app on the same participants. Interrater agreement was assessed using the kappa statistic. A kappa value of 0.41 to 0.60 indicated moderate, 0.61 to 0.80 fair, and 0.81 or more indicated a good agreement [33].

We first tested the app and refined its algorithm in a hospital setting where people with a variety of eye conditions were available. We examined both the patients and their escorts (without eye problems). The purpose was to assess if the algorithm was able to identify referable eye conditions and to refine the procedures that would be followed by CVs during screening.

Following the initial hospital-based testing, we transferred the testing and refinement of the algorithms to a community setting where they would eventually be used in practice. The aim was to assess the usability of the app in identifying people with eye problems and to determine whether the target sensitivity and specificity thresholds could be met.

Interim analysis was conducted after two field tests to determine whether the target sensitivity and specificity had been achieved. For this, we compared referral decisions of the CVs using the app with that of the ophthalmologist as a reference standard. If the target sensitivity and specificity were both not met, data on the decision trees were assessed to determine which specific inputs (questions, measures, or dependencies) needed to be amended, and we made such amendments using our clinical knowledge. The changes were implemented in software, and the validation process was repeated until the sensitivity and specificity targets were met. The accepted end point was determined to be either the targets being met or when all practical combinations had been exhausted.

### Validation Study

#### Study Design and Setting

The validation study was conducted during outreach clinics in selected communities of Trans Nzoia County, Kenya. Most outreach clinics were conducted after church services to provide a broadly representative sample from the community. All consenting participants presenting to outreach centers (irrespective of the type of illness) were eligible to participate. These participants were examined by the same CVs (who had participated in the pretesting), using the Peek Community Screening app, and by one experienced OCO, the reference standard, using standard outreach equipment. Their referral decisions (refer or not) were compared. The study was coordinated by a team from the Kitale Eye Unit.

#### Index Test: Referral Decisions by Community Volunteers Using the Peek Community Screening App

**Figure 2:**
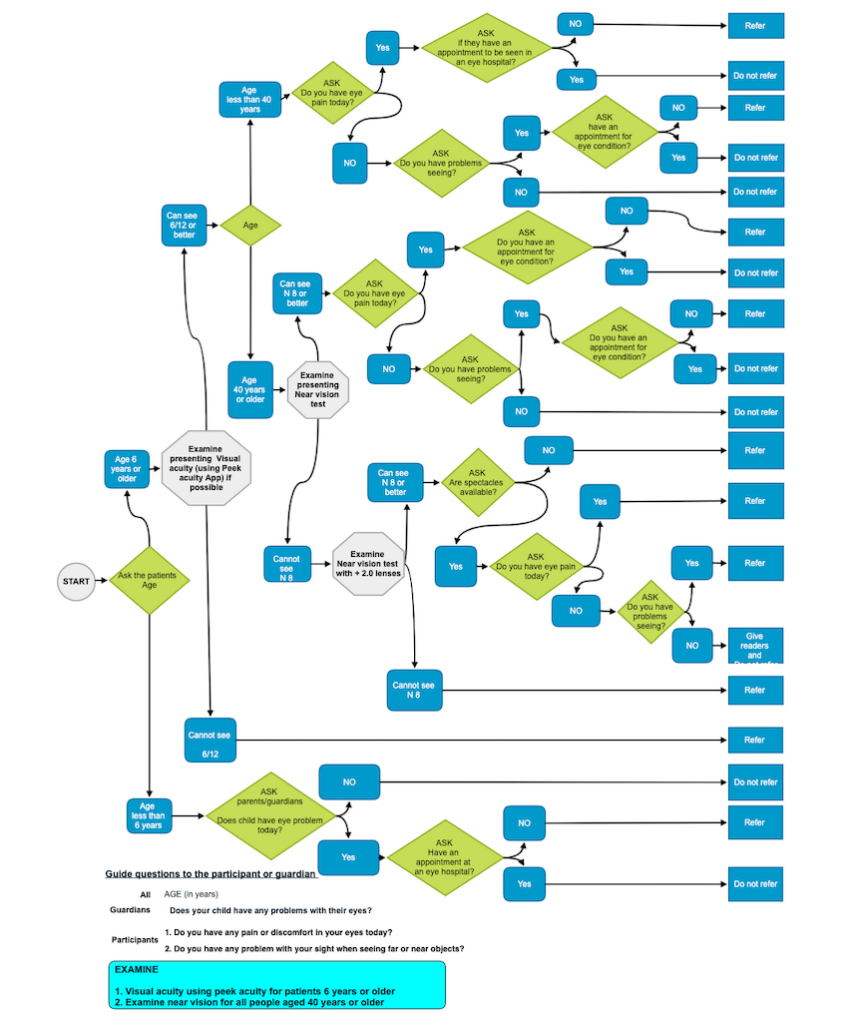
The questions and decisions matrix used in the Peek Community Screening app to generate a referral decision.

In the final test algorithm, users were prompted to ask the following screening questions to the parents or guardian with a child, “Does the child have any problem with their eyes today?” or directly to participant themselves, “Do you have any discomfort or pain in your eyes today?” and “Do you have a problem with your sight when seeing far or near objects?” If the participant was 6 years or older, the app prompts the user to test distance visual acuity using the Peek Acuity app and assess near visual acuity for all people aged 40 years and older at 33 cm using the RADNER reading chart (NeuMed AG) [34]. The distance visual acuity of each eye was measured separately and recorded automatically using the Peek Acuity app [35]. If the distance visual acuity was less than 6/12 in either eye or there was the presence of any self-reported eye pain or discomfort, difficulty seeing distant or near objects, or inability to see N8 on near-vision assessment for those aged 40 years or older, the participant was referred. Any eye problem in children (aged <6 years) as reported by parents or caretakers triggered a referral (Figure 2).

#### Reference Standard: Referral Decisions by Ophthalmic Clinical Officer Using Standard Outreach Equipment

The reference standard was the referral decision by one OCO with 14 years of experience in ophthalmology using standard equipment for outreach. He was familiar with local customs in the setting. The outreach equipment included a Snellen 6-meter vision chart to asses distance vision, RADNER reading chart for near vision, a torch, magnifying loop, i-care contact tonometer, direct ophthalmoscope, retinoscope, trial lens set, and fluorescein stains. Standard slit lamp was not used for assessment because it is not the norm to conduct a slit lamp assessment during outreach in this setting.

#### Study Procedures

Consecutive participants were examined for eye problems by the CVs using the app and, then, by the OCO using standard outreach equipment. The CVs followed the assessment guide and examined visual acuity using the embedded Peek Acuity vision test or near vision using a card when indicated. They entered the participant’s responses in the Peek Community Screening app, where a referral decision was generated automatically. Their decisions were also automatically recorded and uploaded to a dedicated cloud server once the internet connectivity was available.

After the CVs examination, the OCO masked to the decision of the CV, took a detailed history and examined the same participants. Specific information on eye pain, eye discomfort (itching and irritation), tenderness, or eye discharge was collected; vision was assessed as outlined above. A magnifying loupe and torch were used to assess the color of the conjunctiva, the appearance of the pupil, the alignment of the participants’ eyes, the presence of eye discharge, and any lid abnormalities. Direct ophthalmoscopy was used to assess the lens, vitreous, and retina. When indicated, the cornea was assessed using fluorescein and a blue light for corneal ulcers or abrasions. Intraocular pressure was measured using the i-care tonometer. A retinoscope and trial lenses were used to assess refractive errors.

A differential diagnosis for each eye was made for the purpose of management. Recording of the diagnosis followed the Kenyan Ministry of Health classification where the eye could be *normal* (no eye pathology) or any of the following diagnoses: cataract, corneal scars, conjunctivitis, keratitis, uveitis, retinal disease, eyelid disease, presbyopia, other refractive error, foreign body, eye growths, eye injury, and *other*. The OCO selected the applicable diagnosis. All patients were treated as per the OCO’s plan. The OCO recorded their decision and treatment plan on a precoded data collection form.

### Analysis

The primary outcome was the sensitivity and specificity of the CV assessment using the Peek Community Screening app for appropriate referral decisions, compared with the OCO’s recommendation for referral. The minimum target sensitivity was 90% and specificity 75%. Positive and negative predictive values were also estimated. Logistic regression was used to identify whether there was any association between correct decisions being made by CVs and the participants’ age and sex. This was done by using the CV’s referral decisions as the outcome variable with age and sex as exposures, and the analysis was performed separately among those classed as requiring referral or not requiring referral by the reference standard.

We calculated that a sample size of 517 participants was required to estimate a sensitivity to a precision of ±5%, assuming a sensitivity of 90% and that 30.0% (155/517) of participants require referral. Thus, we aimed to recruit this number for the final iteration of the validation.

Data for CVs were downloaded from Peek’s dedicated servers in Excel format, exported to STATA, and, then, cleaned and analyzed. Information from the OCO precoded questionnaire was entered into an Excel database (Microsoft, Seattle, WA, the United States), cleaned, and exported to STATA. Data were analyzed using STATA, version 15.0 (Stata Corp. LP, College Station, TX, the United States) [36]. Age was rounded up to the nearest one year, and the diagnosis was reclassified using the International Statistical Classification of Diseases and Related Health Problems [37].

## Results

This study was conducted between November 2016 and May 2018.

### Interrater Agreement of the Community Volunteers

During the training of the CVs, automated referral decisions were generated by the app for 59 participants, which were used to assess interrater agreement between the reference assessor (lead author) and the CVs. The reference assessor found that 75% (44/59) of the participants required referral compared with 83% (49/59), and 85% (50/59) by CV1 and CV2, respectively. There was 84.8% agreement for referral decisions between the reference assessor and CV1 and 86.4% for CV2; with a moderate kappa of 0.55 and 0.58, respectively.

### Prevalidation of the Peek Community Screening App

One iteration in the hospital and five iterations were tested in the community before arriving at the final version (iteration seven), which was used for the validation study. The changes introduced at each iteration stage and the test performance of the versions are shown in Table 1.

**Table 1:** Sensitivity and specificity of the Peek Community Screening app and the changes introduced at each iteration during validation.

Setting, iteration, and changes introduced			OCO^a^ decision		CV^b^ decision using the Peek Community Screening app						Sensitivity, % (95% CI)		Specificity, % (95% CI)		PPV^c^, % (95% CI)		NPV^d^, % (95% CI)	
					Refer, n		Do not refer, n		Total, n									
**Hospital setting**																		
	**Iteration 1 (enriched sample)**										99.2 (95.4-100**)**		52.4 (29.8-74.3)		92.1 (86.0-96.2)		91.7 (61.5-99.8**)**	
		Ask for the presence of any eye problem (no time limit); distance VA^e^ testing not mandatory for someone with eye problem		Refer		117		1		118								
				Do not refer		10		11		21								
				Total		127		12		139								
**Community setting**																		
	**Iteration 2 (enriched community sample**)										98.8 (96.6-99.8)		66 (51.7-8.5)		93.3 (89.6-96.0)		92.1 (78.6-98.3)	
		*Same question above, in outreach setting with self-selected patients;* ask for the presence of any eye problem (no time limit); distance VA testing not mandatory for someone with eye problem		Refer		250		3		253								
				Do not refer		18		35		53								
				Total		268		38		306								
	**Iteration 3**										97.3 (92.4-99.4)		17.8 (10.5-27.3)		59.8 (52.3-66.9)		84.2 (60.4-96.6)	
		*Introduced mandatory VA testing;* ask for the presence of any eye problem (no time limit); mandatory distance VA testing		Refer		110		3		113								
				Do not refer		74		16		90								
				Total		184		19		203								
	**Iteration 4**										78.4 (72.6-83.6)		75.6 (67.3-82.7)		85 (79.6-89.5)		66.4 (58.3-74.0)	
		*Limited the duration of eye problem to 1 day (today);* ask for the presence of eye problem today; mandatory distance VA testing		Refer		182		50		232								
				Do not refer		32		99		131								
				Total		214		149		363								
	**Iteration 5**										83.7 (77.3-88.9)		61.2 (52.5-69.3)		72.7 (66-78.8)		75.2 (66.2-82.9)	
		*Introduced eye pain instead of eye problem limited to 1 day;* mandatory distance VA testing; asked—any pain in your eyes today? asked—any problem with seeing far or near objects today?		Refer		144		28		172								
				Do not refer		54		85		139								
				Total		198		113		311								
	**Iteration 6**										90.5 (87.1-93.2)		63.3 (57.3-69.0)		77.0 (72.8-80.9)		83.0 (77.3-87.8)	
		*Introduced eye discomfort;* mandatory distance VA testing; asked—any eye pain or discomfort today? asked—any problem with seeing far or near objects today?		Refer		342		36		378								
				Do not refer		102		176		278								
				Total		444		212		656								
	**Iteration 7: Final algorithm**										91.0 (87.7-93.7)		78.1 (71.6-83.6)		88.9 (85.3-91.8)		81.8 (75.5-87.1)	
		Mandatory distance VA testing & near vision for those aged 40+ years; asked—any eye pain or discomfort today? asked—any problem with seeing far or near objects today?		Refer		344		34		378								
				Do not refer		43		153		196								
				Total		387		187		574								

^a^OCO: ophthalmic clinical officer.

^b^CV: community volunteer.

^c^PPV: positive predictive value.

^d^NPV: negative predictive value.

^e^VA: visual acuity.

### Validation Study of the Final Peek Community Screening App

**Figure 3:**
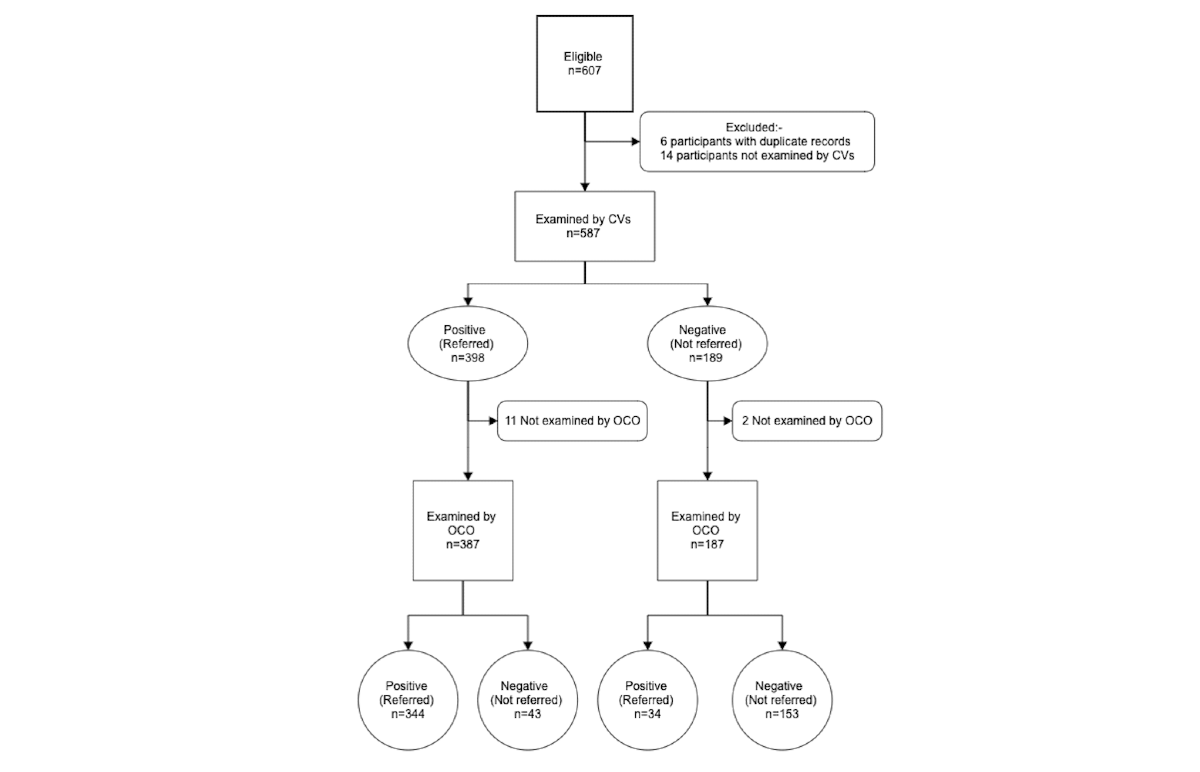
A Standards for the Reporting of Diagnostic Accuracy Studies flow chart for study participants. CVs: community volunteers; OCO: ophthalmic clinical officer.

We included 574 (who had completed the OCO and CV examination and outcome data) out of the potential 607 eligible participants in the analysis of the performance of the seventh iteration of the Peek Community Screening app (Figure 3).

The demographic characteristics of this group are shown in Table 2.

Eye problems that needed referral were diagnosed by the OCO (reference standard) in 65.9% (378/574) of the participants. CVs using the Peek Community Screening app correctly identified 344 out of 378 (sensitivity 91.0%; 95% CI 87.7%-93.7%) participants as having referable eye conditions and 153 out of 196 (specificity 78.1%; 95% CI 71.6%-83.6%) as not. The positive predictive value was 88.9% (95% CI 85.3%-91.8%), and the negative predictive value was 81.8% (95% CI 75.5%-87.1%).

The accuracy of the algorithm varied depending on whether question alone or objectively assessed vision was used. If we used distance visual acuity and assessed near vision for those aged 40 years or older alone, without asking any of the questions about eye pain or discomfort or the question about disturbance in vision, the sensitivity dropped to 42.1% (95% CI 37.0%-47.2%), and specificity was 98.5% (95% CI 95.6%-99.7%).

If we asked about symptoms of eye pain/discomfort and disturbance in vision, with no eye examinations, the sensitivity would be 87.6% (95% CI 83.8%-90.7%) and the specificity would be 79.1% (95% CI 72.7%-84.6%). If the strategy was to refer anyone aged 40 years or older (irrespective of visual acuity of self-reported issues) and those aged under 40 who self-reported either vision problems or eye pain/discomfort, then the estimated sensitivity would be 91.5% (95% CI 88.3%-94.1%) and the specificity would be 77% (95% CI 70.5%-82.7%).

Out of the 196 participants not referred by the OCO (without eye conditions), CVs using the app incorrectly referred (false positives) 21.9% (43/196). There was no evidence to suggest that being incorrectly referred was associated with sex (odds ratio [OR] 0.70; 95% CI 0.35-1.35; *P*=.31) or age (OR 1.00; 95% CI 0.97-1.03; *P*=.86).

Further analysis of these incorrect referrals by the CVs (false positives) showed that the reasons they had been referred were as follows: 7% (3/43) of the participants could not see 6/12 (had VI), 2% (1/43) had both VI and self-reported eye pain or discomfort, 44% (19/43) had self-reported difficulty seeing distant or near objects only, 37% (16/43) had eye pain or discomfort only, and 9% (4/43) complained of both eye pain or discomfort and difficulty seeing distant or near objects. None were because of the near-vision assessment.

Similarly, out of 378 participants who were referred by the OCO (had eye problems), CVs correctly referred 91.0% (344/378). There was evidence (*P*=.003) of a difference in the odds of the CV using the app referring participants by age, with the odds of being referred (if referral was required according to reference standard) higher in those aged 40 or older compared with those under 40 (OR 4.38; 95% CI 1.66-11.59). This was driven by the very high referral rate in the over 40s, with the vast majority being referred both by the OCO and the CV using the app. There was no evidence (*P*=.28) of a difference by sex (OR 1.47; 95% CI 0.72-3.00). Most (25/34, 74%) of the participants classified as false negatives had conjunctivitis (allergic and other; Table 3).

**Table 2:** Age, sex, and visual status of all study participants, those referred by the ophthalmic clinical officer using standard equipment and by community volunteers using the Peek Community Screening app.

Characteristics		Total number (N=574)^a^	Referred by the OCO^b^ (N=378)^c^	Referred using the app (N=387)^c^
**Sex, n (%)**				
	Male	213 (37.1)	135 (63.4)	140 (65.7)
	Female	361 (62.9)	243 (67.3)	247 (68.4)
**Age group, n (%)**				
	<15	252 (43.9)	128 (50.8)	141 (55.0)
	15-29	100 (17.4)	53 (53.0)	55 (55.0)
	30-44	80 (13.9)	57 (71)	53 (66)
	45-59	76 (13.2)	75 (99)	72 (95)
	60-74	52 (9.1)	51 (98)	52 (100)
	75+	14 (2.4)	14 (100)	14 (100)
**Visual acuity (reference), n (%)**				
	Children (vision not assessed)	82 (14.3)	41 (50)	40 (49)
	6/6-6/12	411 (71.6)	256 (62.3)	268 (65.2)
	6/18-6/60	59 (10.3)	59 (100)	57 (97)
	<6/60	22 (3.8)	22 (100)	22 (100)

^a^The distribution of the characteristics of the study participants.

^b^OCO: ophthalmic clinical officer.

^c^Proportions within each characteristic group that were referred by the OCO or community volunteers using the Peek Community Screening app.

**Table 3:** Clinical diagnosis of the participants referred by the ophthalmic clinical officer and referral decisions by community volunteers using the Peek Community Screening app.

Summary of diagnosis	Referral decision by community volunteers using the app	
	Referred (N=344), n (%)	Not referred (false negatives; N=34), n (%)
Cataract	29 (8.5)	0 (0)
Presbyopia	56 (16.3)	2 (6)
Glaucoma	1 (2.9)	1 (1)
Refractive errors	64 (18.6)	2 (6)
Allergic conjunctivitis	117 (34.0)	16 (47)
Other conjunctivitis	44 (12.8)	9 (27)
Corneal disease	2 (0.6)	0 (0)
Retinal disease	5 (1.5)	0 (0)
Eye injury and foreign bodies	1 (0.3)	0 (0)
Uveitis	1 (0.3)	0 (0)
Pterygium conjunctival swellings	10 (2.9)	0 (0)
Chalazion and lid swellings	2 (0.3)	0 (0)
Others	12 (3.5)	4 (12)

## Discussion

### Algorithms Development

We iteratively developed and validated smartphone-based algorithms used by CVs to identify and refer people with eye conditions for services from the community. The standard against which the algorithm was designed and validated was the referral decisions of a trained ophthalmic worker on the same participants.

We predetermined in the study design the acceptable sensitivity and specificity levels to ensure adequate sensitivity to detect people with referable eye conditions in the community and also specificity that is enough not to overburden the system. This was determined as a sensitivity of not less than 90% and specificity not less than 75%.

### Principal Findings

We found that 65.9% (378/574) of the participants enrolled in this study had a referable eye condition based on the examination using standard outreach equipment. This was higher than the prevalence of ocular morbidity found in other studies in Kenya and Rwanda, where the prevalence was 15.2% and 34%, respectively [38,39]. This is likely to be because of differences in the study populations and case definitions used by the studies. We conducted most validation rounds after church when most people could attend an eye check to get a representative sample of the community; however, this may not be an unbiased sample. The case definition for the earlier ocular morbidity study in Kenya excluded minor eye conditions such as pinguecula, which we included [39]. In the Rwanda national survey, only moderate to severe eye symptoms were included, but in our study, all symptoms irrespective of severity were considered [38].

We found that CVs could use the app with moderate interobserver agreement between them and the study ophthalmologist. The accuracy (sensitivity and specificity) of the algorithm was affected by prior duration of the symptoms, the commonality of symptoms and signs across different eye diseases, and the number of signs and symptoms used to generate algorithm. Sensitivity of the algorithm decreased (from 97.3% to 78.4%) with a corresponding increase in specificity (17.8% to 78.6%) when the duration of any eye symptoms was limited to one day from any duration (“Do you have any eye problem today?”). There was a simultaneous increase in specificity (from 61.2% to 63.3%) and sensitivity (from 83.7% to 90.5%) when the presence of pain was expanded to include eye discomfort. Finally, the introduction of near-vision assessment improved the specificity (from 63.3% to 78.1%). It appears that if more signs and symptoms were included in the development of the algorithm, the accuracy could be improved, but the decision to include additional elements had to be balanced with the extra cost of equipment to be used and the level of education and subsequent training requirement of CVs. Overall, the algorithm had to be accurate, acceptable, affordable, and reproducible.

Trained CVs could use the final algorithm to accurately identify and refer people with eye problems (sensitivity 91.0%) and also those without eye disease (specificity 78.1%) in the community. We observed that subjective questions were likely to cause greater variation in responses and, hence, performance of the algorithm.

For example, analysis of the referral criteria used in the algorithm show that self-reported symptoms contributed more to the sensitivity of the algorithm than objective measurement of vision. Had we not asked any of the questions on eye pain or discomfort and the one on disturbance in vision, our sensitivity would have dropped to 42.1%. This would result in missing 219 out of 378 cases determined to be those needing referral instead of the 34 we miss now. In fact, it would be a far better screening test to not do any eye tests at all and just ask for symptoms of eye pain or discomfort and disturbance in vision. This would give us a sensitivity of 87.6% and specificity of 79.1%. Had we just asked the two questions and age, then referred anyone over 40 or who answered yes to either question, we would have got an estimated sensitivity of 91.5% and specificity of 77.0%. The findings suggest that had we excluded the objective measurement, we would have not achieved an acceptable algorithm, unless we had referred everyone older than 40 years. A population-based study in Tanzania found the prevalence of presbyopia among people aged 40 years or older to be 61.7% [40], implying that by referring everyone over 40 years, we could overload the system with false referrals. This concurs with our observation in which participants aged 40 years or older were more likely to be referred by a CV and not by the OCO (false positives).

Similarly, the same self-reported symptoms of eye pain or discomfort and self-reported poor sight contributed to inaccurate decisions from the algorithm. About 81.4% of false positive referrals using the app were from participants self-reporting to have eye discomfort or poor eyesight, whereas only 7% of false positives were because of inaccurate vision assessment. The findings suggest the need for training of the CVs to have skills in basic history taking and examinations. To reduce these false positive referrals, more clinical practice during training could improve CVs’ skills in assessing patients with eye problems. Some studies on performance of CVs [41] suggest a thorough initial training with supportive supervision to improve agreement between assessors. This implies that successful training could aim at certifying CVs who attained minimum agreement (moderate to almost perfect agreement with the reference assessor) before screening the community for eye problems. A further suggestion would be to retrain or even discontinue CVs who do not achieve the desired agreement and include a systematic way to provide continuous assessment on referral appropriateness to maintain posttraining standards.

We found that the participants who were referred by the OCO but not by the CV (false negatives) mostly (25/34, 74%) had ocular surface inflammatory conditions such as allergic conjunctivitis, presbyopia (2/34, 6%), or refractive errors (2/34, 6%; Table 3). We found that most participants with allergic conjunctivitis were correctly referred, suggesting that those identified as false negatives, may have had mild symptoms. This could have resulted from self-reported symptoms that were selectively mentioned to the CV but not to the OCO. Although we did not analyze the severity of allergic conjunctivitis to conclusively classify them as false negatives, other studies have found that some patients who presented with red eyes and allergic conjunctivitis for outpatient consultations had less severe conjunctivitis that could be transient or managed at primary point of contact [42,43].

### Future Improvements

The findings, therefore, suggest the need for a deeper understanding and analysis of allergic eye conditions according to severity. There are suggestions to improve the sensitivity of current algorithm. The first approach is to introduce an assessment for red eyes into the algorithm with integrated images of different types of red eyes to aid in the classification of severity. The second approach is upscaling screeners’ knowledge to distinguish normal and allergic eye disease. The ideal CVs should, therefore, have the skill set to identify VI, referable and nonreferable allergy, and Identification and management of presbyopia. This could, however, require policy change to implement in practice.

Finally, it may be possible to recalibrate the referral criteria for VI based on the capacity of the services, restricting the threshold of referrals to a level that generates referrals of those with more severe VI and lowering this threshold over time as capacity increases to ensure the health system is not overburdened.

As demonstrated, there are multiple factors that affect the performance and acceptance of a guided screening algorithm. These include the subjective and objective inputs in the decision tree. Objective threshold tests such as acuity lead to a binary output (pass or fail), whereas subjective assessments such as self-perception of vision loss have a spectrum of outputs that requires a binary threshold to be derived to progress through the decision tree. Every iteration requires a significant amount of time and resource, making optimization challenging in practice. There is a potential for utilizing Web-based A/B testing techniques currently being used in digital marketing to optimize algorithms more rapidly [44].

### Limitations

There are limitations to be considered in this study. The study was conducted after church services and could have excluded those who did not attend church. Moreover, those who participated may have had a perceived eye problem, which could have resulted in higher prevalence of referable eye conditions and, hence, higher predictive values. There could also be diagnostic uncertainty in the reference standard in this study where an OCO used simple outreach equipment without a slit lamp. The OCOs used as the reference are not available in other health systems and, therefore, the results may be not generalizable to those setting.

### Conclusions

The Peek Community Screening app meets the minimum predetermined criteria. The next step is to incorporate the algorithm into a screening system to assess performance in a health system, to identify people with eye problems, and to link them to primary and secondary centers. We anticipate that more people with eye health needs will be able to access the appropriate level of eye services. More validation studies conducted in different settings and improvement to the existing algorithm may be required. Further research on the performance of the algorithm is needed for specific ages groups (aged 15 years or less, 15-40 years, and those 40 year and older). If acceptable standards are met, it could be of value in determining the population demand for eye services in population-based studies and for being a validated methodology for increasing access to appropriate services in integrated eye health programs.

## Abbreviations

CEHC: Commonwealth Eye Health Consortium
CV: community volunteer
IMCI: Integrated Management of Childhood Illness
mHealth: mobile health
OCO: ophthalmic clinical officer
OR: odds ratio
PHC: primary health care
VI: visual impairment
WHO: World Health Organization
